# Birch pollen—The unpleasant herald of spring

**DOI:** 10.3389/falgy.2023.1181675

**Published:** 2023-05-15

**Authors:** Marianne Raith, Ines Swoboda

**Affiliations:** Competence Center for Molecular Biotechnology, Molecular Biotechnology Section, FH Campus Wien, University of Applied Sciences, Vienna, Austria

**Keywords:** respiratory allergy, birch pollen, pollen proteins, pollen lipids, microbiome, air pollution, climate change, airway epithelial barrier

## Abstract

Type I respiratory allergies to birch pollen and pollen from related trees of the order *Fagales* are increasing in industrialized countries, especially in the temperate zone of the Northern hemisphere, but the reasons for this increase are still debated and seem to be multifaceted. While the most important allergenic molecules of birch pollen have been identified and characterized, the contribution of other pollen components, such as lipids, non-allergenic immunomodulatory proteins, or the pollen microbiome, to the development of allergic reactions are sparsely known. Furthermore, what also needs to be considered is that pollen is exposed to external influences which can alter its allergenicity. These external influences include environmental factors such as gaseous pollutants like ozone or nitrogen oxides or particulate air pollutants, but also meteorological events like changes in temperature, humidity, or precipitation. In this review, we look at the birch pollen from different angles and summarize current knowledge on internal and external influences that have an impact on the allergenicity of birch pollen and its interactions with the epithelial barrier. We focus on epithelial cells since these cells are the first line of defense in respiratory disease and are increasingly considered to be a regulatory tissue for the protection against the development of respiratory allergies.

## Introduction

IgE-mediated respiratory allergies are constantly increasing in industrialized countries (1). The reasons for this increase are still under debate and involve beside the improvement of the health care system that leads to improved diagnosis and greater awareness of the diseases, genetic as well as environmental factors. Environmental factors that have been proposed as causes of higher prevalence are increased allergen exposure resulting from shifts in the geographic distribution or in vegetation time of plants, increased air pollution and a decrease in microbial exposure. The most important plant-derived inhalant allergen source is pollen. Currently, pollen allergy affects about 20%–25% of the European population (2), resulting in impaired quality of life and high socioeconomic costs. In Europe, there are several pollen alert services, mostly provided by the government, which can help affected individuals to avoid outdoor activities on days with high pollen counts. However, it seems that not only the amount of pollen in the air is responsible for the severity of allergic reactions, but that other factors may also have an influence, either on the pollen directly or on the affected individual.

Tree pollen, especially pollen from angiosperms, is well studied from various aspects: e.g., pollen composition and development, the role in the plant as a male gametophyte, the impact on human health as an important allergen source, and the influence of environmental conditions (e.g., air pollution) on pollen, but a multidisciplinary overview is lacking. Therefore, the aim of this review is to combine research from different fields and to provide a comprehensive overview of birch pollen, the major tree pollen allergen source in Central and Northern Europe, its composition, known external influences on its allergenicity, and its effects on the human body, especially on the epithelium of the respiratory tract.

In Central and Northern Europe, the most important tree pollen allergies are caused by birch pollen and pollen from related trees of the order *Fagales*, including the genera *Corylus* (hazel), *Fagus* (beech), *Alnus* (alder), *Quercus* (oak), *Carpinus* (Hornbeam) and *Castanea* (Chestnut), which affect almost 25% of allergic individuals (3). The most dominant pollen allergen of Fagales trees is Bet v 1, the major birch pollen allergen, that cross-reacts with homologous allergens from related trees and usually initiates the sensitization process to these pollens. In Central and Northern Europe, 95% of birch pollen allergic individuals react to Bet v 1 (4), and 60% are solely sensitized to this birch pollen allergen (5). Still, in addition to Bet v 1 five other allergens have been described in birch pollen (see below). Interestingly, pollen from these trees not only affects sensitized individuals through the air, but a number of pollen allergens have homologs in plant foods and cause the so-called pollen-food-syndrome (PFS), formerly known as oral allergy syndrome (OAS) (6).

Most studies focusing on birch pollen as a cause of allergic symptoms, consider the pollen primarily as a carrier of the allergenic molecules Bet v 1 to Bet v 8, while it is often neglected that allergens do not encounter the human respiratory tract in pure form, but together with other pollen-derived proteins (allergenic and non-allergenic), lipids and carbohydrates and other pollen-associated substances, even living organisms (e.g., bacteria or fungi). Single (recombinant) allergens are of great value when it comes to component-resolved diagnostics and may also be beneficial for future therapeutic approaches, but when studying the influence of pollen allergens on airway epithelial cells, the impact of accompanying factors is of immense importance to understand the mechanisms leading to allergic reactions.

## Structure and composition of the pollen grain

### Architecture of the pollen grain

**Figure 1:**
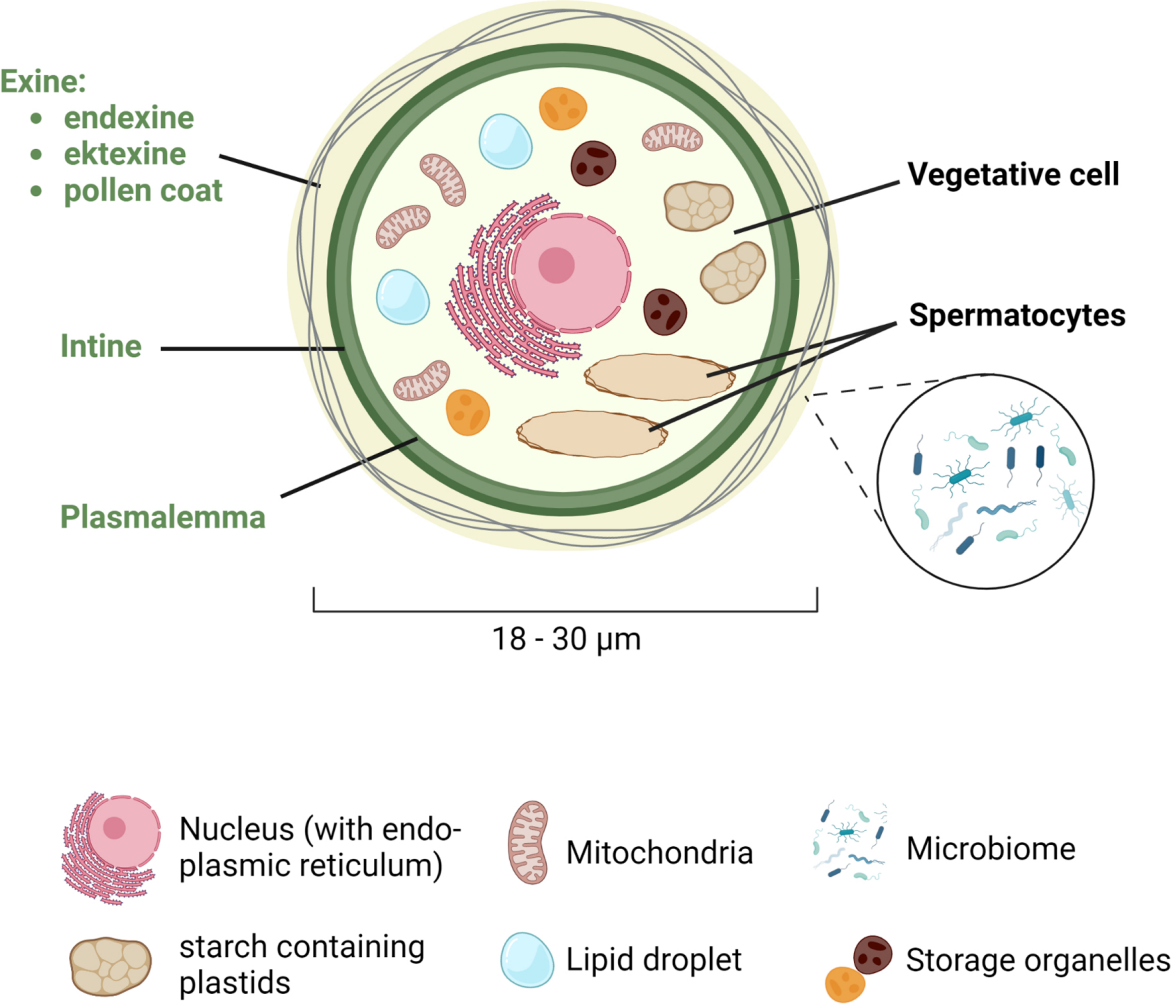
Schematic representation of the structure of the pollen grain. Birch pollen has a diameter of 18 to 30 µm and consists of a large vegetative cell and two smaller spermatocytes. The vegetative cell is surrounded by the plasmalemma, the intine and the exine, the latter being composed of the endexine (towards the intine), the ektexine, and the pollen coat (at the outer most part), which is colonized by the microbiome. In the cytoplasm, the vegetative cell contains a central nucleus with an adjacent endoplasmic reticulum, as well as mitochondria, starch containing plastids, lipid droplets, and other storage organelles. Created with BioRender.com.

Sexual reproduction in wind-pollinated plants starts with the male gametophytes, which are transported through the air to the stigma, which is part of the female birch flower. The pollen itself contains one large vegetative cell and two smaller spermatocytes (Figure 1). The spermatocytes have their own plasmalemma and are thus separated from the vegetative cell. In addition to the two spermatocytes, the vegetative cell has a large central nucleus with an adjacent endoplasmic reticulum, mitochondria, starch containing plastids and many storage organelles. They are rich in proteins, carbohydrates and lipids, often contained in specialized organelles, called lipid droplets.

The pollen grain is surrounded by a series of envelops forming a thick wall, starting with the plasmalemma that surrounds the cytoplasm, followed by the intine and the exine, which is then coated by various lipids such as sterol esters or triacylglycerides (the pollen coat). While the intine consists mainly of cellulose (fibrillar cellulose and hemicellulosa), pectins and associated enzymes important for the germination and growth of the pollen tube, the exine consists of sporopollenin, an extremely stable structure. The exine itself has two layers: the endexine towards the intine and the ektexine (consisting of a foot layer, a columella layer, and the tectum) towards the pollen coat. The lipids in the pollen coat (also known as pollenkitt) protect the pollen from external influences such as UV light (7) or dehydration (8), but also from pathogens. The pollen coat is also a habitat for various symbiotic microbes, which form the pollen microbiome. In contrast to the continuous lining of the intine, the exine also has one or more thinner areas, called apertures that facilitate water and gas exchange and from where the pollen tube germinates. The number, morphology, and position of apertures vary greatly among species (9). In dicotyledonous plants, such as birch, three apertures are predominant. In addition to their function in the germination process, the apertures also play an important role in adaption of the pollen to volume changes due to desiccation and hydration (10).

### Lipids in the pollen

As expected from the multilayered structure of the pollen wall and intracellular structures, a high number of fatty acids are found in the pollen grain. The lipids that form the phospholipid-bilayer of the plasma membrane and the typically large endoplasmic reticulum in the vegetative cell are synthesized *de novo* in the chloroplast stroma. The most abundant fatty acids of these cellular membranes are octadecadienoid (linoleic) and hexadecenoic (palmitic) acids (11). As in most biological membranes in photosynthetic plants, glycerophospholipids dominate, although the polar head groups can be highly variable. In pollen membranes, choline, ethanolamine, serine, glycerol, and inositol are the most abundant (12).

In addition, a highly stable and chemically resistant layer of sphingolipids is found in the membrane of the plasmalemma and the endoplasmic reticulum, which play an important role in the response to biotic and abiotic stress, such as drought (13). Other important lipids in plant pollen grains are glycoceramides (mainly in intracellular membranes) and galactolipids (mainly in plastids), the latter also playing an important role in the response to abiotic stress.

As mentioned above, cytoplasmic lipid droplets are also present in pollen. They consist of a monolayer of phospholipids and a core of triacylglycerides, which make up the bulk of lipid droplets (14). Lipid droplets are known to have structural and/or metabolic functions and thus represent reserves of energy-rich metabolites and other components for future metabolic needs. Lipid droplets increase during pollen development and are transported from the pollen grain to the pollen tube, where they are involved in pollen germination, penetration of the stigma and pollen tube growth (15).

It is known that among the immunomodulatory components present in pollen, lipids play an important role. In this context it has been shown that lipid-derived fatty acids can either directly interact with allergens such as Bet v 1 (16) or they can influence the immune response by their interaction with innate lymphocytes, such as natural killer T (NKT) cells (17). It has indeed been shown that polar lipids, diacylglycerols, free fatty acids, and triacylglycerols can be internalized by dendritic cells and can be presented by CD1 molecules to NKT cells, which release cytokines such as IL4 that promote the differentiation of Th0 lymphocytes into Th2 cells (18). Two of the most important forms in which lipids can reach the airways and modulate the immune response, are lipopolysaccharides (LPS) released by bacteria, which will be discussed later in this review, and so-called pollen-associated lipid mediators (PALMs). Under the term PALM, mediators with structural and functional homology to eicosanoids are summarized. Behrendt et al. showed that substantial amounts of PALMs are released during hydration of pollen grains (19). In the pollen, PALMs appear to be involved in the stress response to pathogens or heavy metal exposure (20, 21). Due to their similarity to eicosanoids, PALMs can induce maturation and migration of dendritic cells and trigger Th2 cell polarization that then contributes to the development of allergy in pollen-exposed tissues of predisposed individuals (22).

### Proteins and enzymes in the pollen

**Figure 2:**
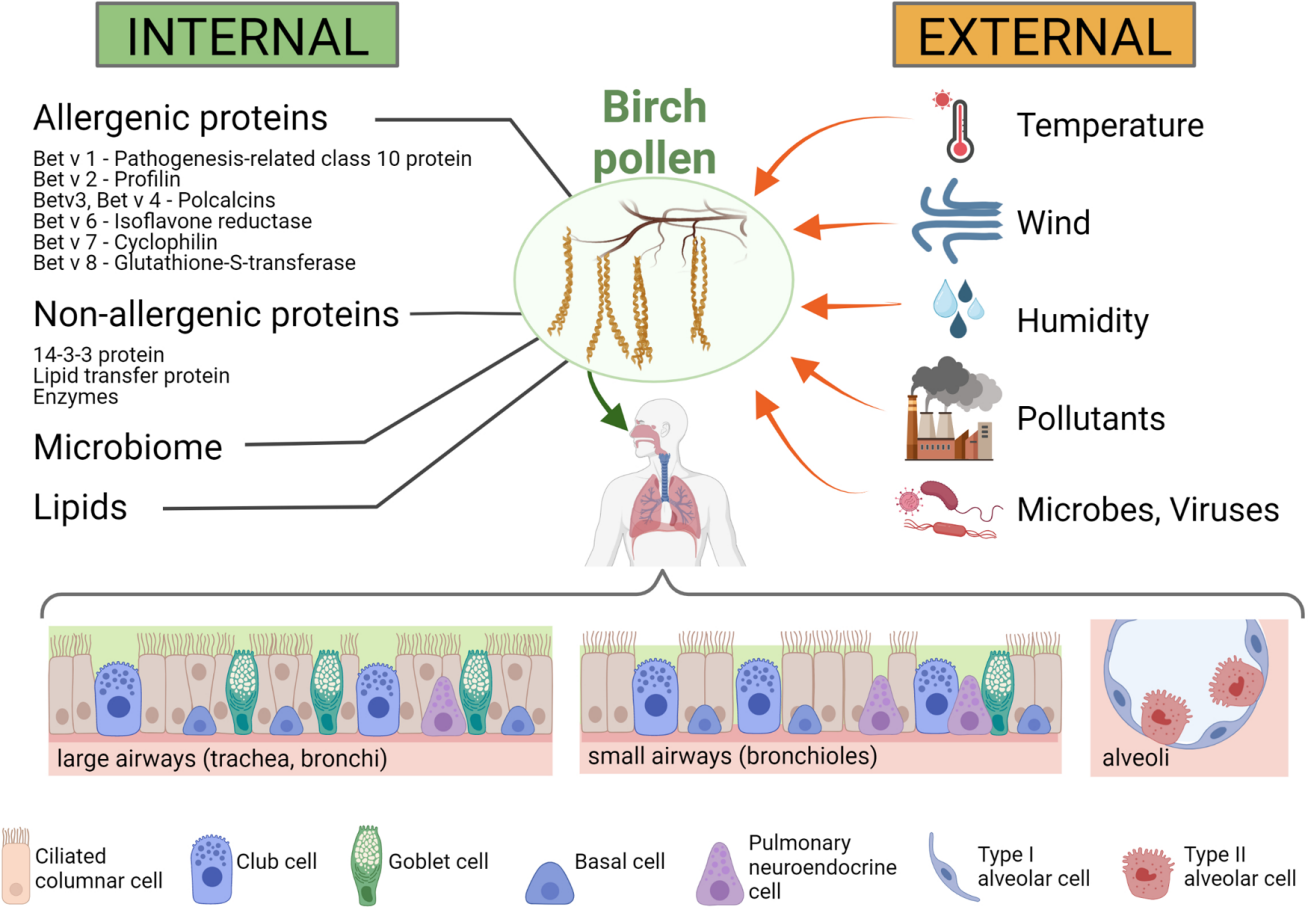
Internal and external influences on birch pollen and the pollen's interaction with the human respiratory tract. Internal influences on the immunomodulatory effects of birch pollen (**left panel**): Birch pollen contains a number of allergenic proteins, such as Bet v 1 (a pathogenesis-related class 10 protein), Bet v 2 (a profilin), Bet v 3 and Bet v 4 (both polcalcins), Bet v 6 (an isoflavone reductase), Bet v 7 (a cyclophilin) and Bet v 8 (a glutathione-S-transferase), but also non-allergenic immunomodulatory proteins such as 14-3-3 protein, lipid transfer proteins and a number of enzymes. In addition, also lipids are present in high amounts in birch pollen, and microbes (such as proteobacteria or actinobacteria) (84) and viruses (such as idaeovirus or cherry leaf roll virus) (85) are found in the pollen coat. External influences on birch pollen and their immunomodulatory effects (**right panel**): Meteorological influences such as temperature, wind and humidity can affect the immunomodulatory activity of the pollen and the pollen load, but other biotic (e.g., microbes and viruses) and abiotic (e.g., air pollutants) factors can also affect the allergenicity of the pollen. Lower panel: Respiratory cells that interact with the birch pollen. The cellular composition of the airway epithelium changes throughout the respiratory tract, with the large airways (trachea, bronchi) having higher number of goblet cells than the small airways (bronchioles) or the alveoli. Created with BioRender.com.

Mature pollen is usually metabolically inactive and has a rather small transcriptome. Genes expressed in pollen contribute to cell wall metabolism as well as carbohydrate and energy metabolism. As in any eukaryotic cell, cytoskeletal proteins and proteins for signal transduction are also expressed. Here we attempt to provide an overview of the most relevant allergenic and immunologically relevant non-allergenic proteins in birch pollen known to date (Figure 2, left panel). However, we are aware that this is ongoing research and therefore this list needs to be constantly expanded.

## Allergenic proteins

### Bet v 1 and Bet v 1-like protein family

Bet v 1 is the major allergen in birch pollen and belongs to the pathogenesis-related protein class 10 (PR-10), which are highly conserved proteins of small size of about 160 amino acids and a molecular mass of ∼17 kDa with a similar structure (23). More than 100 proteins of the PR-10 class are known today, among them also many other aeroallergens and food allergens that are known to be highly IgE cross-reactive and are thus regarded as important panallergens. In general, PR-10 proteins are suggested to participate in defense mechanisms and play a protective role in the plant. However, PR-10 proteins are also constitutively expressed in some parts of the plant (e.g., pollen) and could also have other functions, such as in plant growth or development (23). In 1989, the first Bet v 1 cDNA was cloned by immunoscreening of a pollen cDNA library using sera of birch pollen allergic individuals (24). Later, several Bet v 1 isoforms have been identified, which differ only by a few amino acids (25, 26), but display variable IgE reactivities, with Bet v 1a showing the strongest IgE binding capacity (27). Crystallographic analyses revealed that Bet v 1 has a hydrophobic cavity that can bind different ligands depending on the Bet v 1 isoform (28). One of the most interesting Bet v 1 ligands is quercetin, a glycosylated flavonoid, because this siderophore plays an important function in iron binding and thus influences the allergenicity of Bet v 1 (29–31). It has been shown that Bet v 1 in the absence of iron induces T cells to elicit a Th2 response, which is not the case when quercetin binds Fe^3+^ and ligates to Bet v 1 (31). The explanation for this could be a change in the structure of Bet v 1, as it has been shown that interaction with the ligand increases the volume of the hydrophobic pocket, causing a structural change that could affect the uptake and processing of the protein (32, 33).

### Profilin (Bet v 2)

Another plant panallergen present in birch pollen is Bet v 2, a member of the profilin family, which is expressed in pollen, seeds and fruits of almost all plants (34). Profilins are actin-binding proteins, which can also bind poly-L-proline or phosphoinositide and thus play an important role in many signaling pathways responsible for cell growth, apoptosis, vesicular transport or lipid metabolism (35). Profilins from different sources have a molecular mass of ∼15 kDa and a highly conserved structure that consists of a compact beta sheet in the center surrounded by several alpha helices (36). In particular, the IgE-binding regions are highly conserved [as reviewed in (37)], resulting in strong IgE cross-reactivity between most plant profilins. Because of the high cross-reactivity, patients sensitized to Bet v 2 also react to other plant sources such as pollen from grasses or weeds (38) or plant foods such as pear, cherry, and celery (39). Interestingly, sensitization rates vary widely within Europe: while 2%–12% of birch pollen allergic patients from Northern European countries show IgE reactivity against Bet v 2, the sensitization rate in patients from Central and Southern Europe is up to 50% (40, 41).

### Polcalcins (Bet v 3 and Bet v 4)

Beside Bet v 1 and Bet v 2, the polcalcins Bet v 3 and Bet v 4 can be regarded as the third group of panallergens in birch pollen. Polcalcins are ubiquitous pollen proteins with molecular masses of 24 kDa (Bet v 3) and of 7–8 kDa (Bet v 4). They are thought to play a role in pollen germination and, as the name “polcalcin” suggests, also in the regulation of calcium levels in pollen, which is underlined by their conserved structure with two (Bet v 4) or four (Bet v 3) EF-hand motifs (42, 43). Both, Bet v 3 and Bet v 4 have been shown to bind IgE, but their sensitization rates are rather low with 15% for Bet v 3% and 21% for Bet v 4 among birch pollen allergic individuals (44). Even though sensitization rates in birch pollen allergic individuals are low, sensitization to polcalcins can have a strong impact on the sensitized patients due to the high cross-reactivity between polcalcins from different species.

### Isoflavone reductase (Bet v 6)

Isoflavone reductase Bet v 6, formerly known as Bet v 5, has a molecular mass of ∼35 kDa and was identified as an allergen in 12%–32% of birch pollen allergic individuals and shows cross-reactivity to isoflavone reductases present in certain fruits and vegetables among them also orange, strawberry, persimmon or zucchini (45). Isoflavone reductase has also been described in Japanese cedar, where unlike Bet v 6, it is a major allergen causing seasonal pollinosis (46). In legumes, isoflavone reductases are well studied and are known to have various functions, especially in the defense against pathogens (47); however, the function in birch pollen is not fully understood (48). In general, in pollen, isoflavone reductases seem to play a role in the germination, the pollen tube growth and the pollen-stigma recognition (49).

### Cyclophilin (Bet v 7)

Bet v 7, a member of the cyclophilin A family, has a molecular weight of ∼18 kDa and was identified as a birch pollen allergen in 1995 (50, 51). Bet v 7 exhibits IgE cross-reactivity to other plant cyclophilins, e.g., from hazel pollen, meadow grass pollen or tomato (51). In contrast no cross-reactivity to allergenic cyclophilins from fungal species was detected (51). In plant pollen, cyclophilin has been shown to be induced in response to biotic and abiotic stressors, such as heat, drought or in response to fungi (52).

### Glutathione-S-transferase (GST, Bet v 8)

The most recently discovered allergen in birch pollen is Bet v 8, a protein homologous to glutathione-S-transferase (GST) with a molecular mass of ∼27 kDa. GST has been shown to bind IgE, with a sensitization rate of ∼13% in a cohort of Austrian birch pollen allergic patients (44). Interestingly, GST has also been reported as an allergen in house dust mites (53), cockroaches (54), *Alternaria alternata* (55), and wheat (56). However, in pollen allergic individuals with a known IgE reactivity to Bet v 8, no cross-reactivities between Der p 8 (the GST from house dust mites) and Bet v 8 were found (44). In the plant, GSTs are involved in various plant functions, such as detoxification of xenobiotics, secondary metabolism, growth and development, and most importantly, protection against biotic and abiotic stress (57).

## Non-allergenic proteins

### 14-3-3 protein

The phosphoserine/threonine binding protein 14-3-3, with a molecular weight of 25–30 kDa, interacts with lipoxygenases and in this way affects the levels of pollen-associated lipid mediators (PALMs). Together with other non-allergenic proteins, it has been shown to be upregulated in urban pollen as compared to pollen from rural areas (58, 59). It was suggested that pollutants might be involved in the upregulation of 14-3-3 and other non-allergenic proteins and that this might contribute to the higher allergenic potential of urban as compared to rural pollen, whose allergen content remained unchanged (58).

### Lipid transfer protein

Birch pollen also contains lipid transfer proteins belonging to the pathogenesis-related protein 14 (PR-14) group, but in contrast to LTPs present in ragweed or Parietaria pollen or in plant foods such as peach (60), LTP from birch pollen has not yet been identified as an allergen. In Arabidopsis, LTPs have been shown to play a role in systemic resistance signaling (61, 62) or in cuticular wax accumulation (63, 64). However, LTPs also play a central role in pollen and seed development (65) as well as in fruit development (66) and seed germination (67). Probably the most important role of LTPs is their action in response to biotic stress (e.g., to fungi) (68) and their putative response to abiotic stress (e.g., to drought) in plants (69).

### Enzymes in the pollen

In a multi-approach analysis, it was shown that among the proteins easily released from birch pollen and thus present in *Betula verrucosa* pollen extracts were endogenous proteases as well as proteases of bacteria (70). Using a proteomics-based approach McKenna et al. identified in total 42 proteases, which belong to the catalytic classes of serine-, cysteine-, aspartic-, threonine-, and metallo-proteases (70). The endogenous proteases are usually important for the germination process and are activated when pollen grains hydrate on a compatible stigma (71). However, pollen can also hydrate when they come into contact with the mucosa of the respiratory epithelium (72). For other allergenic pollen it has been shown that proteases can either be allergens themselves (73) or that they can damage as non-allergenic proteins the epithelial barrier and thus allow allergens to enter the tissue, leading to sensitization or, in already sensitized individuals, provocation of an allergic response (74). A further way, how pollen enzymes can contribute to allergic reactions is shown by NADPH oxidases. These enzymes produce reactive oxygen species (ROS) in the airway epithelium, which could thus be a factor that promotes allergic sensitization or exacerbates allergic reactions (75).

## The microbiome of the pollen

The pollen coat contains lipids and sugars that are an ideal source of nutrients for microorganisms such as bacteria or fungi, and indeed pollen usually carries several microorganisms on its sticky pollen coat. These microorganisms seem to have an impact on the allergenicity of birch pollen, and higher bacterial diversity has been associated with higher amounts of Bet v 1 and PALMs produced by birch pollen (76). On the other hand, bacterial diversity appears to be affected by air pollutants, with NO_2_ levels being negatively and O_3_ levels being positively correlated with bacterial diversity (76). Pollutants thus influence the pollen microbiome, which then impacts the expression of allergenic proteins and PALMs in the birch pollen.

It is known that microbial exposure, either to microbes from the environment or to the endogenous microbiome influences immune responses and has also an effect on the development of allergic diseases. Microbial exposure is actually regarded as an important factor in the protection against allergic diseases, as it has been shown that the so-called “farm effect”, namely the exposure to microbes in early infancy, can modify allergy susceptibility. For example, it has been shown that the prevalence of childhood asthma in urban areas was significantly higher than in children who grew up in traditional agricultural environment, although the genetic background was similar (77). The “farm effect” may be explained, at least in part, by exposure to high levels of lipopolysaccharides (LPS), a component of the cell wall of gram-negative bacteria and of bacterial DNA (78). In addition, a Finnish birth cohort study and a German study both showed that the risk of asthma was lower in non-farm households when the composition of the microbiome in these households was similar to that in households of farmers. This suggests that microbial diversity and the presence of specific microbes has a protective effect, rather than the number of bacteria (79). However, LPS has to be considered as a molecule with a dual role that, in addition to its antiallergic function, may also promote the development of allergy. Kaur et al. showed that the protective effect of LPS depends on the presence or absence of the immune-modulatory cytokine GM-CSF (80).

The environmental and the endogenous microbiome can interact, and the environmental microbiome can change the composition of the host microbiome and may therefore also influence the development of allergic diseases. This is in line with the observation that in asthmatic individuals different levels of bacteria (e.g., increased levels of proteobacteria) and a reduced diversity of the lung microbiome were observed as compared to healthy individuals (81). Interestingly, the microbial composition of skin exposed to urban green spaces was shown to resemble the microbiome in soil, whereas the microbiome in nasal samples resembled the microbiome in air (82), thus the environment has a direct influence on the diversity of the microbiome. However, it is not known whether environmental microbes can survive on the skin or mucous membranes and whether they can replicate (83).

## External influences on birch pollen

Plants are exposed to a variety of natural stress factors, including too much or too little water or sunlight, different intensities of UV radiation, too low or too high temperatures, or mechanical factors such as the wind, but also anthropogenic stress factors such as particulate or gaseous air pollutants (Figure 2, right panel).

### Pollutants

Plants are very important for all aerobic organisms because they absorb carbon dioxide (CO_2_) and produce oxygen (O_2_) in all green parts of the plant by photosynthesis, providing vital oxygen, but also removing the important greenhouse gas CO_2_ from the atmosphere, thus counteracting global warming. However, besides their importance for the production of O_2_ and the removal of CO_2_, the leaves of some trees, such as *Betula pendula*, are also able to capture particulate matter (PM) and thus have a positive impact on the removal of pollutants, especially in urban areas (86). Diesel exhaust particles (DEP), produced by incomplete combustion of diesel fuel in motor vehicles, were also found in the coating of birch pollen (87, 88). DEPs consist of a carbon core on which organic chemical components (CO, NO, NO_2_, SO_2_, hydrocarbons) and heavy metals are deposited (89). More than 80% of all DEPs belong to the ultrafine particle range, i.e., they have a diameter of less than 0.1 µm and can therefore easily enter the respiratory tract, including the lower airways (90, 91). In addition, these ultrafine particles have been shown to penetrate the lipid bilayer of alveolar epithelial cell membranes and to be translocated transcellularly by diffusion (92) and are thus capable of causing airway inflammation and tissue damage [as reviewed in (93)]. Even worse, DEPs could also be a vehicle for birch pollen allergens, a similar effect has already been described for Lol p 1, an important grass pollen allergen from *Lolium perenne* (94).

Air pollution (e.g., PM) and high ozone (O_3_) concentrations have also been shown to increase the levels of stress proteins in plants and also in pollen, and since some of these stress proteins are allergens (e.g., Bet v 1), the allergenicity of pollen also increases (95, 96). In addition, it has been suggested that elevated O_3_ levels not only lead to the formation of birch pollen with increased Bet v 1 content, but also alter PALM composition, ultimately leading to a higher proinflammatory potential of the pollen (95). Moreover, it has already been shown that nitration of Bet v 1.0101 by NO_x_, a group of gaseous air pollutants, leads to oligomerization of the allergen (97), thus increasing its allergenicity (98). Interestingly, Stawoska et al. recently showed that urbanization and air pollution also affect the secondary structure of Bet v 1, by inducing a decrease of regular α-helix and β-structures and an increase of β-turns together with anti-parallel β-sheet structures. It can be expected that such modifications will most likely also affect the protein's functions and probably even its allergenicity (99). In addition to these rather indirect effects of pollutants on the pollen, pollutants can also act directly on pollen by increasing the fragility of the exine, which then facilitates the release of substances, among them allergens, into the environment (100).

### Meteorological events and climate change

#### Temperature

The main drivers of greenhouse gases are increased CO_2_, methane, and NO_x_ concentrations in the atmosphere, which lead to an increase in temperature, also known as global warming. The increase in temperature due to global warming also affects the composition and diversity of plant communities (101). While some tree species have problems adapting to the changing environmental conditions, *Betula* species appear to be adaptable and can even survive under drought and high-temperature stress (102). The rise in temperature can induce an earlier onset of pollen seasons (103), which has been described for many sites in Europe in case of birch pollen (104, 105). In general, analysis of data from 34 years of observation (1959–1993) showed that spring events in Europe were advanced by an average of 6 days, while autumn events were delayed by 4.8 days, resulting in an overall extension of the annual growing season by 10.8 days (106). However, earlier onset of the pollen season could also lead to an interruption of the season due to unfavorable weather conditions in late winter/early spring [as reviewed in (107)]. This in turn could lead to an increase in allergens, as a delay in pollen release (e.g., due to lower temperatures) allows pollen to spend more time in the anthers and thus to mature longer, resulting in higher pollen allergen content (108). However, the opposite can also be the case: when immature pollen is dispersed, the allergen content is lower, because it has been shown that Bet v 1 is only expressed in mature birch pollen shortly before pollination (109). Immature pollen does not mature after dispersal because it dries up immediately after release from the anthers (110) and lacks the water required for biochemical processes.

#### Wind

Most allergenic plants are wind pollinated (e.g., trees of the order *Fagales)*, for this, they have developed special strategies to increase the probability of pollination: They develop small, dried pollen with good aerodynamic properties that allow them to be transported over distances of several hundreds of kilometers (111–113). In the past few centuries, birch trees have also become increasingly popular as decorative plants, especially in parks and public places, which has led to an increase in pollen counts from these trees (114). In addition, elevated CO_2_ concentrations are also known to boost plant growth and thus lead to increased pollen production. In general, the content of pollen grains in the air during pollination can be quite high, ranging from 1,000 to 10,000 pollen grains per 1 m^3^. In the case of wind-pollination, however, it is the size that matters. While the relatively small birch pollen with a diameter of about 18 to 30 µm can be transported over very long distances, grass pollen (e.g., from timothy grass) with an average diameter of 22 to 122 µm can only be transported over shorter distances (115, 116). While intact pollen grains are usually found in the coarse fraction of particulate matter (i.e., sizes greater than 10 µm), pollen fragments can also be found in the fine particulate matter fraction (i.e., smaller than 2.5 µm), the latter being able to penetrate the alveolar cells of the lungs (117).

#### Humidity and thunderstorms

Global warming also leads to a change in precipitation patterns. The allergenic proteins normally found in the pollen protoplast are released during rehydration (118). Thus, for the birch pollen allergens Bet v 1 and Bet v 2, it has been shown that they are found in the anhydrous state in the pollen cytoplasm, usually in close proximity to ribosome-rich areas, but are released from the apertures within minutes after rehydration and are subsequently found on the entire pollen surface (118–120). In addition to precipitation under normal conditions, climate change also effects the frequency and intensity of thunderstorms and tropical cyclones (121). In particular, thunderstorms are thought to induce the rupturing of pollen due to an osmotic shock and the subsequent liberation of a high concentrations of allergenic particles of small size that can reach the lower airways and are then responsible for the extreme asthma events associated with thunderstorms (122, 123).

## Interactions of birch pollen with the human airways

As mentioned above, Bet v 1 and many other allergens have been shown to be quickly released within minutes after contact of the pollen with a humid environment, such as the mucosa (119). It is thought that the moist surface of the mucosa induces the release of allergens, but also of non-allergenic proteins and other bioactive molecules from the pollen. In order to initiate sensitization process, allergens then need to get into contact with sub-epithelial immune cells. For this, they have to cross the epithelial cell layer, which protects the body from external noxious agents. The fact that the disruption of this epithelial barrier promotes allergic sensitization, has led to the concept of the epithelial barrier hypothesis (124, 125). Disintegration of the epithelial barrier can occur due to a variety of causes, including proteolytic activities of the allergen itself, as in the case of the major house dust mite allergen Der p 1 (126) or, as mentioned earlier, due to non-allergenic proteases present in the allergen source or in the associated microbiome, but also due to a concomitant exposure to other risk factors, such as viruses, bacteria, or toxins such as cigarette smoke (127, 128), to name only a few.

The airway epithelium is composed of different cell types, including ciliated columnar, goblet and basal cells (Figure 2, lower panel). In addition, there are pulmonary neuroendocrine cells and secretory Club cells (formerly known as Clara cells). Cell types vary in different parts of the airways: in the large airways (from the nose to the trachea and the larger bronchi), there are predominantly ciliated columnar epithelial cells, whereas in the bronchioles and terminal bronchioles there is a mixture of non-ciliated and ciliated cells, and in the alveoli (the respiratory tissue) the majority of cells are non-ciliated alveolar type I and II cells. While the main function of the goblet cells is mucus secretion, the ciliated cells are crucial for the mechanical removal of foreign particles from the lungs through the coordinated movement of their apical cilia, and the main function of the alveolar cells is gas exchange. The combined action of the mucin-producing secretory goblet cells with the movement of the cilia of ciliated cells results in efficient removal and expulsion of foreign material, also known as “mucociliary clearance” (129). However, it is important that mucus production is tightly regulated, as it was shown that in respiratory allergies, mucus release from goblet cells is stimulated by IL-13 [a cytokine produced from Th2 cells and considered as a central mediator in allergic asthma, as reviewed in: (130)] leading to hypersecretion of mucus, which may contribute to disease exacerbation in affected individuals. The non-ciliated, secretory epithelial Club cells have the capacity of self-renewal and can differentiate into ciliated cells. Club cells play a key role in the protection of the epithelial cell layer by secretion of substances lining and protecting the bronchioles of the airways and by detoxification of inhaled foreign substances (131).

Although the cellular composition of each section of the respiratory tract is different, the term “united airway hypothesis” was introduced, since upper and lower airways represent a unified morphological and functional unit with common structures, such as the ciliary epithelial cells, the glands, and the goblet cells. Pathophysiological and clinical evidence for the “united airway hypothesis” is the observation that the pathological process of one section of the airway also affects the function of the entire respiratory system, such as allergic rhinitis in the nose and the manifestation of asthma in the lower airways (132, 133). The fact that one trigger (e.g., birch pollen) can cause two symptoms (e.g., rhinitis and asthma) can be explained by the finding that immune response to allergens are the same in the upper and lower respiratory tract involving the activation of T cells, IgE class-switching in B cells, activation of mast cells, basophils and eosinophils, and the expression of chemokines and cytokines (134–136).

As already demonstrated, the epithelium is not only a mechanical barrier, but it has been shown to play an active role in both innate and adaptive immune responses. Epithelial cells recognize allergens with different kinds of pattern recognition receptors (PRRs) and respond [as reviewed in: (137); or shown in: (138)] by expression of pro-inflammatory chemokines and cytokines, such as CCL-5, IL-8, IL-1ß, IL-18, IL-25, TSLP and IL-33 (139). Lipids and carbohydrates released from the pollen and from microorganisms present on pollen are also recognized by PRRs and can thus contribute to the pro-inflammatory immune response (140). Interactions between inhaled allergens and allergen-associated lipids also occur at the air-liquid interface of the airways, as type II epithelial cells produce surfactant that covers epithelia in the airways and is known to be itself composed of a high proportion of lipids (∼90%), such as phosphatidylcholine and phosphatidylglycerol as well as proteins (∼10%) (141). Due to differences in the surfactant composition and in the surface activity between asthmatic patients and non-asthmatic individuals, it was suggested that the surfactant composition could play an important role in the pathogenesis of allergic asthma (142, 143). Whether the altered surfactant composition is the reason or the cause for allergic asthma is not yet known, but it has been shown that different compounds of the surfactant counteract allergic reactions (144). Furthermore, it has been shown that the lipid-binding allergen Bet v 1 can be transported through the nasal epithelial cells by lipid rafts in a caveolar-dependent manner (145). Once allergens have crossed the epithelial barrier, they are taken up, processed, and presented by antigen-presenting cells (APCs), such as dendritic cells (DCs). However, allergens can also activate DCs directly, as DCs can also reside directly beneath the airway epithelium in the conducting airways and extend their dendrites through the tight junctions of the bronchial epithelial lining into the lumen of the airways (as reviewed in: (146). Cytokines released by the epithelial cells provide an environment that supports DC activation and initiates their migration to the lymph nodes, where they present the processed allergen MHC-II to naïve T cells and induces their polarization into Th2 cells. The origin of IL-4 required for Th2 polarization is still not known. Either basophils or NKT cells are proposed as sources of IL-4. The Th2 immune response then initiates the class-switch of B cells to IgE-producing plasma cells.

Interestingly, it has already been shown that the allergenic potential of a protein depends on specific characteristics of the peptide presented in the HLA-complex. For example, for Bet v 1, it has been shown that more than 60% of individuals sensitized to birch pollen recognize the same T cell epitope and that such allergens with immunodominant peptides show higher sensitization rates than others (147). In contrast, allergens lacking an immunodominant T cell epitope, such as the Bet v 1-homolog from apple, Mal d 1, or from celery, Api g 1, do not seem to induce allergy sensitization (33).

Although the mechanisms of both phases, the sensitization as well as the effector phase are well known, it is still not clear how allergens can activate the immune system and whether different allergens interact in a similar way with the airway epithelial cell layer. Therefore, further studies at the molecular level are needed to understand the pathophysiology of type I hypersensitivity reactions.

## Conclusion and challenges

In summary, IgE-mediated respiratory allergies to tree pollen, such as birch pollen, are a major burden, especially in regions with continental climate, and the prevalence of these respiratory allergies is constantly increasing. Various factors affect the allergenicity of birch pollen: external factors such as air pollution or climate change, exposure of trees and their pollen to biotic and abiotic stress, resulting in a shifted or prolonged pollen season, or the expression of stress response factors and an altered microbiome on their surface. These factors also influence the interaction between birch pollen and respiratory epithelial cells and immune cells. Understanding the mechanism of this altered reactivity is important for future effective treatments or for establishing preventive measures for respiratory allergies. However, the study of birch pollen allergy in the context of environmental conditions and epithelial cells presents a number of challenges.

• Investigating the composition of birch pollen can be challenging, as their composition can be highly variable, depending on the location of the sampling, e.g., in urban or rural areas, but also depending on weather conditions and may not be comparable from one location or one day to another.
• To study the effects of external conditions and birch pollen on the human respiratory tract, either patient studies are performed, animal models are used, or human epithelial cell cultures are established. However, all these methods raise additional issues, such as ethical concerns, insufficient patient numbers, problems in excluding interindividual differences, problems in transferring data obtained in animal (e.g., mouse) models to humans, or the artificiality of the cell culture system. The solution probably lies in the combination of several methods and in an interdisciplinary approach.
• *In vitro* studies of environmental factors affecting birch pollen and the respiratory epithelium often have a weakness in the experimental setup, as it is difficult to mimic external influences such as air pollution in an experimental setup. For example, it is challenging to incubate cells of the respiratory epithelium with particulate matter or gaseous pollutants because stimulation of these cells is only possible within narrow limits regarding toxicity so that their viability is not impaired.

In conclusion, the study of birch pollen allergy in terms of environmental conditions and epithelial cells presents a number of challenges. However, by using different approaches, a better understanding of the mechanisms underlying this disease can be gained, which may contribute to the development of more effective treatments and prevention measures.
